# Isolation of Thylakoid Membrane Complexes from Rice by a New Double-Strips BN/SDS-PAGE and Bioinformatics Prediction of Stromal Ridge Subunits Interaction

**DOI:** 10.1371/journal.pone.0020342

**Published:** 2011-05-26

**Authors:** Jinzhen Shao, Yubo Zhang, Jianlan Yu, Lin Guo, Yi Ding

**Affiliations:** 1 State Key Laboratory of Hybrid Rice, Department of Genetics, College of Life Sciences, Wuhan University, Wuhan, China; 2 Department of Biochemistry, College of Life Sciences, Wuhan University, Wuhan, China; 3 College of Life Sciences, Hubei Normal University, Huangshi, China; University of South Florida, United States of America

## Abstract

Thylakoid membrane complexes of rice (*Oryza sativa* L.) play crucial roles in growth and crop production. Understanding of protein interactions within the complex would provide new insights into photosynthesis. Here, a new “Double-Strips BN/SDS-PAGE” method was employed to separate thylakoid membrane complexes in order to increase the protein abundance on 2D-gels and to facilitate the identification of hydrophobic transmembrane proteins. A total of 58 protein spots could be observed and subunit constitution of these complexes exhibited on 2D-gels. The generality of this new approach was confirmed using thylakoid membrane from spinach (*Spinacia oleracea*) and pumpkin (*Cucurita spp*). Furthermore, the proteins separated from rice thylakoid membrane were identified by the mass spectrometry (MS). The stromal ridge proteins PsaD and PsaE were identified both in the holo- and core- PSI complexes of rice. Using molecular dynamics simulation to explore the recognition mechanism of these subunits, we showed that salt bridge interactions between residues R19 of PsaC and E168 of PasD as well as R75 of PsaC and E91 of PsaD played important roles in the stability of the complex. This stromal ridge subunits interaction was also supported by the subsequent analysis of the binding free energy, the intramolecular distances and the intramolecular energy.

## Introduction

As one of the most important cereal crops and model organisms, the rice, *Oryza sativa* L., is widely cultivated in the Southeast Asia. The yearly rice consumption per capita in China is more than 100 kg. A better understanding of photosynthesis mechanisms is expected to facilitate the engineering of more adaptable and abundant staple crops. Chloroplast thylakoid membranes, which disperse throughout the stroma, are sites of oxygenic photosynthesis in green algae and higher plants. The thylakoid membrane system contains many hydrophobic integral membrane proteins and hydrophilic membrane-associated proteins. These include four multiprotein complexes: the photosystem I (PSI), PSII, ATP-synthase and cytochrome b_6_/f complexes. Together, these complexes operate as a sunlight-driven electron transport chain that generates ATP[1]. In the PSI complex, PsaC and two other extrinsic subunits (PsaD and PsaE) constitute the stromal ridge subcomplex. These three subunits are located on the top of PsaA and PsaB, and the PSI reaction center. The stromal ridge was suggested to have an important role in docking electron acceptors for the PSI complex[2], [3].

Different proteomic techniques have been applied in the studies of chloroplast subfractions, the thylakoid membrane, the lumen and semifluid matrix. Two dimensional isoelectric focussing/sodium dodecyl sulfate-polyacrylamide gel electrophoresis (2D-IEF/SDS-PAGE) was successfully used in separating chloroplast lumen proteins in *Arabidopsis thaliana* [4], peripheral thylakoid proteins in *Pisum sativum* [5], and chloroplast proteins in *Oryza sativa* [6]. However, the classical 2D-IEF/SDS-PAGE has its limitation in identifying the chloroplast integral membrane proteome despite of its power in resolving a large number of soluble and peripheral membrane proteins. The 2D-IEF/SDS-PAGE lysis buffer containing chaotropes urea and thiourea is not effective in extracting membrane proteins from lipid membranes and keeping the proteins in solubilized forms in the aqueous environment. This is because that hydrophobic membrane proteins tend to aggregate during IEF and this presents a problem for transferring the proteins from gel matrices of the IPG strips to SDS gels in the second dimension[7]. One-dimensional (1D) SDS-PAGE combined with mass spectrometry (MS) analysis has also been applied in studies of thylakoid integral membrane proteins[8]. Despite of the identification of some membrane proteins in these studies, a substantial of information regarding protein interaction is missing in the previous proteomic analysis.

Blue native (BN)/SDS-PAGE was firstly developed by Schagger and von Jagow[9] to directly reveal the constitution of membrane protein complexes in the native form. This technique has been widely used to study chloroplast protein complexes in *Spinacia oleracea* [10], *Nicotina tabacum* [11], *Pisum sativum* [12], *Arabidopsis thaliana* [13], *Sugar beet* [14], and *Hordeum vulgaris* [15].

A previous statistical analysis has shown that integral membrane proteins are more difficult to identify by MS than soluble ones, because the transmembrane domains of these proteins frequently lack the cleavage sites for trypsin[16]. Increasing membrane protein quantity could overcome the difficulty in some instances[17], [18]. Thus, developing a method to improve protein abundance in BN/SDS gel could not only display the membrane protein interaction patterns, but also increase the likelihood of identifying these integral membrane proteins.

In this article, we introduced a new method named as “Double-Strips BN/SDS-PAGE” which improves protein abundance on the gels. Using this method, a total of 58 protein spots were separated from the thylakoid membrane preparations of rice chloroplast. When the method was used to separate thylakoid membranes from *Spinacia oleraceaand* and *Cucurita spp.*, a total of 70 and 92 spots were resolved on the BN/SDS-gels, respectively. Using our method, nine protein complexes, including holo- and core- PSI, PSIIcore, Cytochrome b6/f, CP43-less of PSII core, F_0_-F_1_ and F_1_-ATP synthase, monomeric, dimeric, trimeric forms of light harvest complex II (LHCII) in rice were screened in a gel. Peripheral subunits PsaC, PsaD, and PsaE, which are constituents of the stromal ridge complexes, were also identified. In order to predict the interaction among the stromal ridge subunits PsaC, PsaD, and PsaE, we used the homology modelling method combined with molecular dynamics (MD) simulations to refine the stromal ridge model. Computation modelling showed that the salt bridges play important roles in the interactions between the residues R19 of PsaC and E168 of PsaD as well as between the residues R75 of PsaC and E91 of PsaD.

## Results and Discussion

### Solubilization of membrane complexes with optimal detergent-protein ratios

In order to select an optimal detergent-protein ratio for dissolving membrane complexes, 50 µg aliquots of thylakoid membrane (50 µg chlorophylls, ∼500 µg protein) were dissolved in 50 µl of different concentration of DDM lysis buffer (0.5, 1.0, 2.0, 3.0 and 4.0% [w/v] in 750 mM aminocaproic acid/50 mM Bis-Tris, pH 7.0). The supernatants were electrophoresed under non-denature conditions. The efficiency of protein solubilization with different DDM/protein ratios for membrane complexes could be evaluated, according to the patterns of protein complexes resolved on the 1D BN-PAGE, we also evaluated the solubilizing efficiency of TritonX-100 and NP-40 at a detergent-protein ratio of 4∶1.

**Figure 1:**
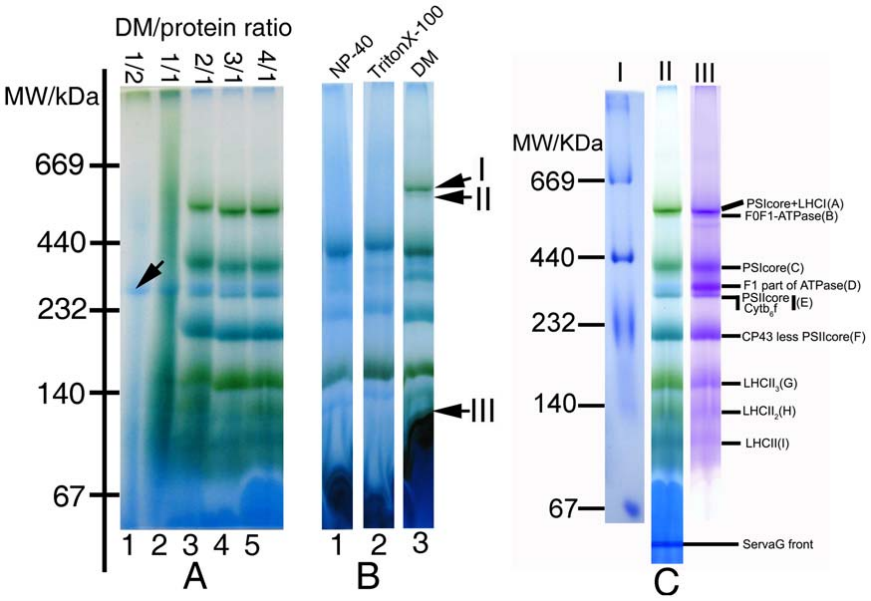
Pattern of thylakoid membrane complexes separated from rice by 1D BN-PAGE. **A**: The lanes 1, 2, 3, 4, and 5 were loaded with preparations of 50 µg chlorophyll(∼500 µg protein) dissolved in 50 µl of 0.5, 1.0, 2.0, 3.0, and 4.0% DDM lyses buffers. **B**: Each slot of lanes 1, 2, and 3 was loaded with the supernatants of 50 µg chlorophyll(∼500 µg protein) dispersed corresponding to 50 µl 4% NP-40, TritonX-100, and DDM lyses buffers. **C**: Lane I was loaded with high MW markers dissolved in 50 µL 4.0% DDM lyses buffers and electrophoresed in the same condition with Lane II. Lane II was loaded with the supernatant of 50 µg chlorophyll dispersed in 50 µL 4.0% DDM lyses buffers and scanned directly after electrophoresis, and lane III was the same as lane II and scanned after stained by Coomassie Blue, nine membrane complexes were labelled on the left of Coomassie-stained gel.

Only one hydrophilic ATPase complex was observed on the 1D BN-gels when a DDM-protein ratio of 1/2 (g/g) was used in the preparation (lane 1, Figure 1A). This suggests that the amount of DDM used was not enough to achieve the critical solubilization concentration (CSC) required for disrupting a membrane system into a predominantly micellar dispersion [34]. The lane 2 of Figure 1A showed tailing of protein bands in electrophoresis. This indicated that the detergent-protein ratio of 1/1 (g/g) could partially break apart the thylakoid membrane into “lipid-DDM-protein” and “lipid-DDM” micelles. However, when the detergent-protein ratios were elevated to 2/1, 3/1, 4/1 (g/g), the higher amounts of DDM could better resolve the membrane proteins in the form of “protein-detergent” micelles, as shown by distinct separation of the membrane complex into individual proteins on the gel (Lane 3, 4 and 5, Figure 1A). Furthermore, the protein separation pattern generated at the DDM-protein ratio of 4/1 showed the best efficiency in resolving the protein complexes (lanes A∼I, Figure 1C). At the same time, we also evaluated the resolving efficiency of NP-40 and Triton-X 100 at a detergent-protein ratio of 4/1 (g/g). As shown in Figure 1B, the PSI holo-complex (arrow I), F_0_F_1_-ATPase complex (arrow II), and monomeric form of LHCII (arrow III), could be well separated when the thylakoid membrane was dissolved at a detergent: protein ratio of 4/1. However, these complexes were not resolved by NP-40 and Triton-X 100 (Lane 1 and 2, Figure 1B). Therefore, all further experiments were carried out using DDM as the detergent, and samples were processed at DDM/protein ratio of 4/1. Under this condition, nine protein complexes could be exhibited on 1-D BN-PAGE gels. Molecular mass of these complexes was determined to be in the range from 108 to 568 kDa (Figure 1C and Table 1) according to the HMW markers. Due to the presence of Coomassie dyes and chlorophyll during gel electrophoresis, most of the protein complexes became visible without staining as blue or blue-green bands (lane II, Figure 1C). When the parallel lane of 1D BN-gels were stained by Coomassie blue R-250, a total of nine protein complex bands could be clearly observed with an enhanced visibility, especially for band D (F_1_- ATP synthase), and the identities of these thylakoid membrane complexes are labelled to the right of the panel (Lane III, Figure 1C) and listed in table 1.

**Table 1:** Apparent MW of thylakoid membrane complexes from rice (*Oryza sativa* L.).

Bands	Complexes	MW(kDa)
A	PSI holo	568
B	F_0_F_1_-ATPase	548
C	PSI core	379
D	F_1_-ATPase	327
E	PSIIcore+Cytb_6_/f	309
F	PSIIcore CP43 less	231
G	LHCII_(3)_	165
H	LHCII_(2)_	134
I	LHCII	108

### Comparison of the resolving efficiency between the Double-Strips and the traditional BN/SDS-PAGE method in rice thylakoid membrane

**Figure 2:**
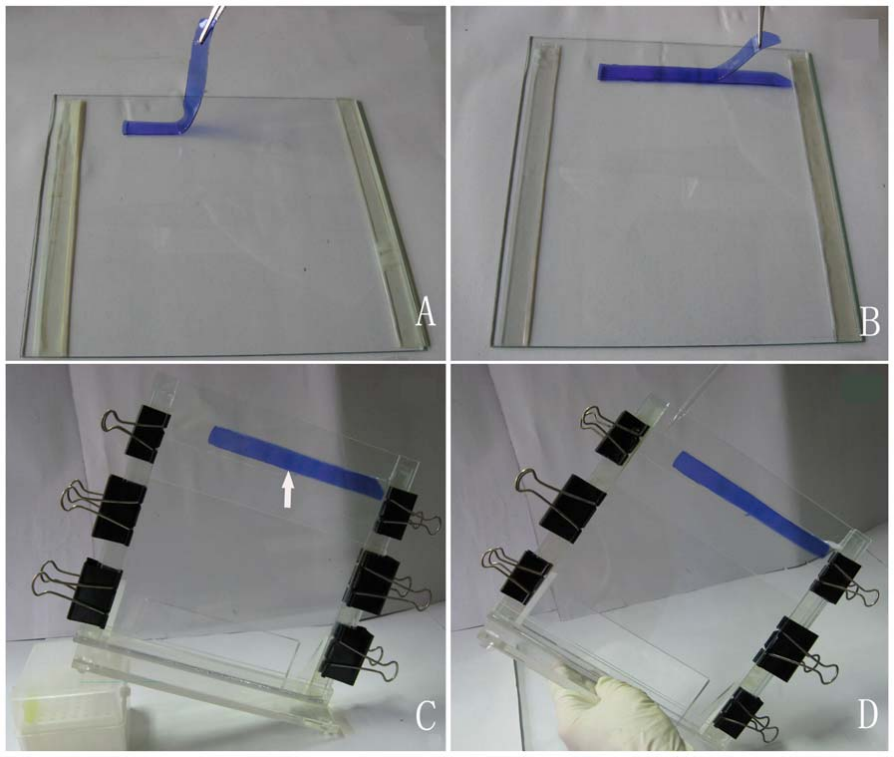
The operation of a novel “Double-Strips 2D BN/SDS-PAGE” transfer technique from the first dimension to the second. **A**: One strip of 1D BN-gel lane was placed in the glass plate after denaturing. **B**: The other identical BN-gel strip was covered to the previous according to the aligned protein complexes, and then the glass plate was overlapped on the gels. **C**: The cassette was placed in skew form avoiding air bubble produced, the agarose was poured in the space between the BN-gel and stacking gel (just the half of all volume, as arrow indicated) after acrylamide was polymerized. **D**: The remaining space was subsequently filled with agarose until the previous solution has been concreted.

In the traditional BN/SDS-PAGE method (e.g. single strip BN/SDS-PAGE), the amount of sample loaded in 1D BN-gels was limited by the slot volume, and furthermore, just only one lane strip was transferred to the second dimensional, some constituent subunits of the membrane complexes, especially for those expressed in low level, could not be exhibited on 2D SDS-gels. In order to circumvent the obstacle, a new gel-based method “Double-Strips BN/SDS-PAGE” was developed in this study for the purpose of increasing the abundance of protein spots separated in 2D SDS-gels. The main workflow of this method was described in the method section and illustrated in Figure 2.

**Figure 3:**
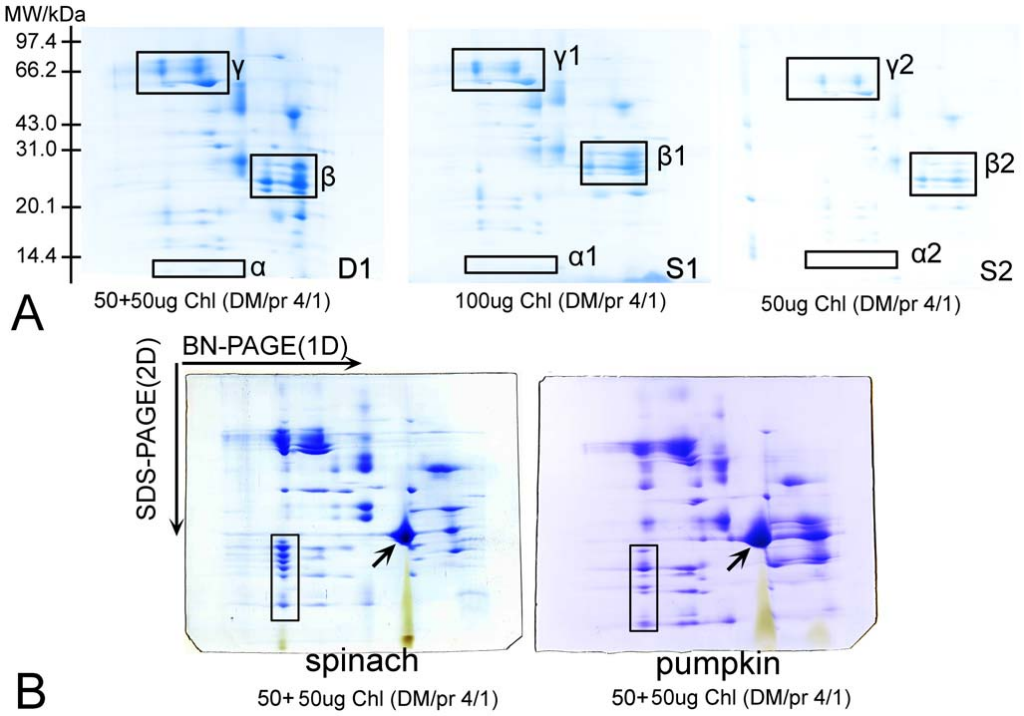
Resolution comparison between “single-strip BN/SDS-PAGE” and "double-strips BN/SDS-PAGE” with membrane proteins. **A**: Comparison of 2D gels pattern between “Single-strip BN/SDS-PAGE” and “Double-strips BN/SDS-PAGE” in rice. (**D1**): The pattern of “Double-Strips BN/SDS-PAGE” with two 1D BN-Gel lane strips each loaded with 50 µg chlorophyll dissolved at DDM/protein ratio of 4/1. (**S1**): The pattern of “Single-Strip BN/SDS-PAGE” with one 1D BN-Gel strip loaded with 100 µg chlorophyll dissolved at DDM/protein ratio of 4/1. (**S2**): The pattern of “Single-Strip BN/SDS-PAGE” with one 1D BN-Gel strip loaded with 50 µg chlorophyll dissolved at DDM/protein ratio of 4/1. (Parts of regions α, β and γ in the panels of 2D SDS-gels represent the obvious difference between these methods). **B**: The pattern of the thylakoid membrane proteins from *Spinacia oleracea* and *Cucurita spp.* separated by “Double-Strips BN/SDS-PAGE”. The amounts of chlorophyll and detergent/protein ratios were labelled in the panels.

To compare the resolving efficiency of “Double-Strips BN/SDS-PAGE” and “traditional BN/SDS-PAGE”, we initially performed separation of membrane proteins from rice with the same lane strips of 1D BN-gel, which each slot was loaded with equal sample (50 µg chlorophyll, ∼500 µg protein). To ensure the reliability of the experiment results, the electrophoresis parameters, SDS-gel dimension, and Coomassie stain were all controlled at the same conditions. In traditional BN/SDS-PAGE, only one strip of 1D BN-gel was transferred, so it obtained a low intensity of protein spots and inferior resolving efficiency of protein pattern (panel S2, Figure 3A). When two lane strips of 1D BN-gel were transferred in superimposed manner, the protein spots resolved on 2D gels were much more intensive (panel D1, Figure 3A and Figure S1). From D1 to S2 of Figure 3A, we can see that the difference between them in protein resolution and number of protein spots is mainly caused by the amount of sample loaded, and similarly, the protein spots detected in the 100 µg single-strip 2D gels (panel S1, Figure 3A) were still increased in number and intensity than the 50 µg single-strip 2D gel (panel S2, Figure 3A). When with the same protein loading using traditional and Double-Strips methods were compared, the former had still lesser resolving efficiency than the latter although both of the methods analyzed equal proteins. Obviously, the method of Double-Strips BN/SDS-PAGE displayed higher resolving efficiency than traditional method (panels S1 and S2, Figure 3A) in the study.

Specifically, protein spots on Double-Strips 2D-gels were more visible than those separated by the traditional Single-Strip BN/SDS-PAGE. For example, the low molecular weight proteins (MW<21 kDa) could not be observed on single-strip 2D SDS-gels since its low abundance in native complexes (as indicated in square region α1and α2, panels S1 and S2, Figure 3A). However, we could excise and identify four protein spots on Double-Strips 2D-gels in the corresponding region, as indicated in square region α of panel D1 (Figure 3A). Two of these spots were identified as cytochrome b559 alpha subunits, with calculated MW of 9439 Da. Thus, double-strips method has the ability to exhibit small hydrophobic peptide due to superimposing two lane strips. As to the middle (21 kDa<MW<43 kDa) and high molecular weight proteins (43 kDa<MW<97 kDa), these protein spot intensities also increased on the double-stripes 2D-gels (as indicated in square regions β_,_ and γ in Figure 3 A). Seeing that double-strips BN/SDS-PAGE could significantly increase the abundance of subunits of photosynthetic complexes on 2D-BN/SDS gels, therefore, this technique could facilitate the subsequent protein spot excisions and MS detection.

To clearly demonstrate the effect of DDM-protein ratios on the resolving efficiency, we separated rice thylakoid membrane by loading ∼1000 µg of proteins prepared at a DDM-protein ratio of 2/1 in an 1D “traditional BN/SDS-PAGE” (one strip). Interestingly, protein patterns obtained exhibited tailing in horizontal dimension (panel S3, Figure S2). This suggested the importance of optimized detergent/protein ratio in sample preparation at the first dimension.

Overall, the abundance and intensity for protein spots of membrane proteins resolved by “Double-Strips BN/SDS-PAGE” were superior to traditional BN/SDS-PAGE (D1>S1>S2, Figure 3A). Each sets of experiments were repeated four times (Figure S1 and Figure S2) in our study, all results indicated that this new method is advantageous in terms of reproducibility and resolving efficiency for the membrane protein preparations.

We also attempted to separate the thylakoid membrane of dicotyledons using this novel Double-Strips BN/SDS-PAGE method. A total of 70 and 92 spots could be observed on 2D SDS-gels of samples prepared from *Spinacia oleracea* and *Cucurbita spp.*, respectively (Figure 3B). The constituted subunits patterns of each complex in dicotyl displayed on 2D SDS-gels were similar to that of *Oryza sativa* L. Interestingly, minor difference of protein spot intensities in some complexes could be observed between dicotyl and monocotyledon. For instance, the light harvest complex II from dicotyledon was more easily observed comparing with that of *Oryza sativa* L. (as arrow indicated in Figure 3B). These indicate the abundance of the light harvest complex may be higher in the dicotyledons.

### Identification of thylakoid membrane proteins from *Oryza sativa*

**Figure 4:**
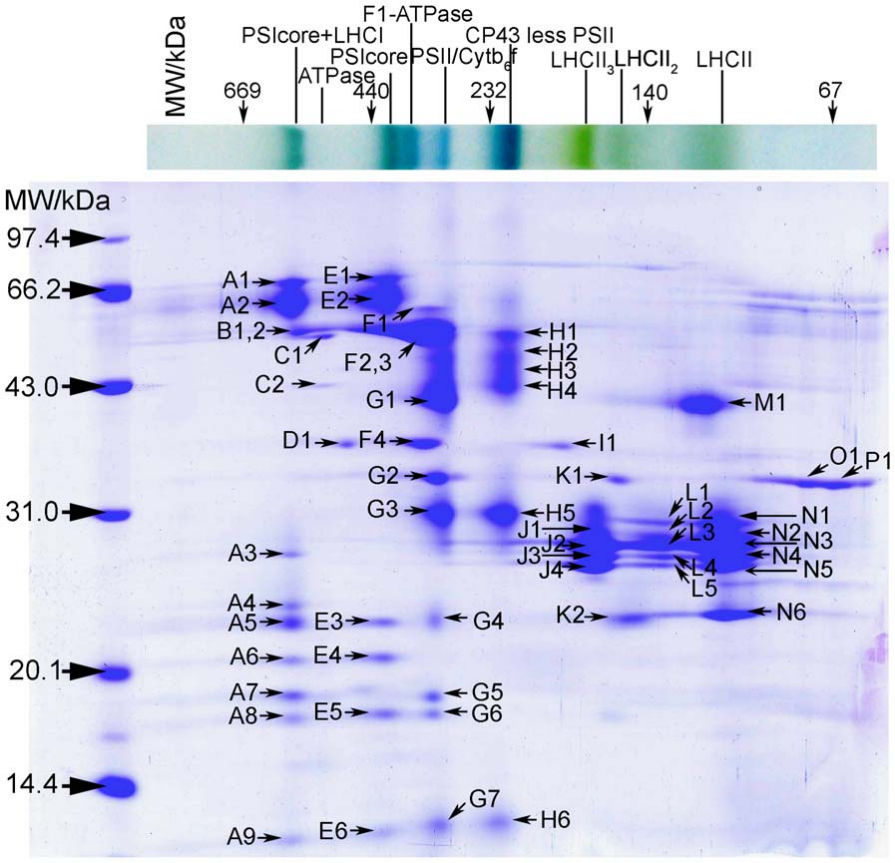
The constituent subunit pattern of thylakoid membrane complexes in rice (*Oryza sativa* L.) resolved using “Double-Strips BN/SDS-PAGE” (Each protein spot was marked with capital letter plus Roman numeral, which was corresponded to the spot mark in **Table 2**.)

BN/SDS-PAGE has been widely applied to separate the thylakoid membrane proteins in dicots spinach[10], pea[12], arabidopsis[13] and monocots barley[16]. The complex pattern of rice thylakoid membrane displayed in the first BN-gels in our study was similar to the previous reports. However, subunit constitutes of rice thylakoid membrane resolved by Double-Strips BN/SDS-PAGE exhibited a little difference, mainly having higher resolving efficiency than previous reports[35]. The protein spots (constituent subunits) were manual excised from 2D SDS-gels (Figure 4) and digested by trypsin. Peptide mixtures were processed by MALDI-TOF or MALDI-TOF/TOF and these enable us to distinguish the identities of most thylakoid membrane proteins (Table 2, Figure S3), a total of 58 protein spots have been identified, in which 48 protein spots identified by MALDI-TOF and 10 protein spots by MALDI-TOF/TOF.

**Table 2:** Identification of thylakoid membrane proteins in rice (*Oryza sativa* L.) by MALDI-TOF.

Spot No.	Accession number	Protein	Mascot	Protein	Gravy	TMHMM/	Pep	Seq.
		description	Score	MW (Da)		HMMTOP	No.^d^	Cov.(%)^e^
A1^a^	gi:11466786	PsaB	108	82622	0.106	9/11	14	31
A2^b^	gi:20146741	PsaA	135	83042	0.249	9/11		
A3	gi:49388156	chlorophyll a/b-binding protein type III precursor	57	29191	−0.035	−/3	5	41
A4 a	gi:47497405	Putative 20 kDa chaperonin, chloroplast	63	20320	0.038	−/−	7	48
A5	gi:125558131	PsaE	78	15440	−0.357	−/−	6	60
A6	gi:115477831	PsaD	78	22134	−0.384	−/−	7	39
A7	gi:34393861	hypothetical protein	53	14615	−0.73	−/−	3	37
A8	gi:125527224	OsI_03231	41	5256	−0.36	−/−	2	98
A9	gi:115455189	Os03g0736600	67	25089	−1.325	−/−	7	31
B1 a	gi:20146763	ATPase alpha subunit	82	55202	−0.052	−/1	16	36
B2 a	gi:11466794	ATPase beta subunit(CF1)	110	54037	−0.102	−/−	25	56
C1	gi:56966762	Rubisco Chain A	84	53401	−0.275	−/−	9	24
C2	gi:11466853	NADH dehydrogenase subunit 7	175	45827	−0.132	−/−	18	62
D1	gi:108864048	Fructose-bisphosphate aldolase	80	41808	−0.201	−/1	10	45
E1 b	gi:20146741	PsaA	135	83043	0.249	9/11		
E2	gi:11466786	PsaB	69	82622	0.106	9/11	7	19
E3	gi:125558131	PsaE	72	15440	−0.357	−/−	6	56
E4	gi:115477831	PsaD	62	22134	−0.384	−/−	5	33
E5 b	gi:131225	PSI-L	108	22197	0.251	2/3		
E6	gi:149390673	dynamin 2b	50	14316	−1.479	−/−	4	35
F1	gi:11466784	ATPase alpha subunit(CF1)	130	55687	−0.074	−/1	16	34
F2	gi:11466784	ATPase alpha subunit(CF1)	74	55687	−0.074	−/1	16	37
F3	gi:552857	ATPase beta subunit(CF1)	135	53977	−0.079	−/−	21	52
F4	gi:115472339	ATPase gamma subunit	68	40081	−0.091	−/−	9	34
G1	gi:11466771	CP43	97	52214	0.274	7/7	15	34
G2	gi:110288946	cytochrome f	77	33909	−0.082	1/1	10	43
G3	gi:11466770	D2	99	39776	0.363	6/6	13	28
G4	gi:11466819	Cytochrome b6	75	24281	0.57	4/5	7	33
G5 a	gi:115472727	Cytochrome b6-f complex iron-sulfur subunit	80	24211	−0.08	1/1	7	31
G6 b	gi:8131597	Qb(Segment)^c^	256	14347	−0.062	2/2		
G7	gi:11466807	Cytochrome b559 alpha chain	55	9439	0.019	1/1	4	37
H1	gi:109156612	CP47	124	56353	0.096	5/5	18	37
H2	gi: 42795571	CP47	108	53572	0.079	5/5	14	23
H3	gi:109156612	CP47	173	56353	0.096	5/5	22	49
H4	gi:109156612	CP47	144	56353	0.096	5/5	17	40
H5	gi:57834096	D2	79	17182	0.157	1/1	8	55
H6	gi:11466807	cytochrome b559 alpha chain	52	9439	0.019	1/1	3	37
I1 b	gi:218155	chloroplastic aldolase	69	42122	−0.227	−/1		
J1	gi:218172	LHCII type I	80	28081	0.098	−/3	8	52
J2 b	gi:115438250	LHCII type I	85	27535	0.09	−/3		
J3	gi:115472785	LHCII type III	100	28817	0.025	−/2	9	48
J4	gi:115453971	LHCII type II	85	28534	−0.087	−/3	6	49
K1	gi:110288946	Cytochrome f	141	33909	−0.082	1/1	13	48
K2 b	gi:115458738	CP24	65	27043	0.062	−/2		
L1 b	gi:115472753	CP29	80	31330	−0.09	−/3		
L2 b	gi:115472753	CP29	79	31330	−0.09	−/3		
L3	gi:115472785	LHCII type III	77	28817	0.025	−/2	7	34
L4	gi:115453971	LHCII type II	75	28534	−0.087	−/3	6	47
L5	gi:115472785	LHCII type III	118	28817	0.025	−/2	11	62
M1	gi:226683	CP43	53	52100	0.282	7/7	8	18
N1 b	gi:115472753	CP29	182	31330	−0.09	−/3		
N2	gi:125533937	Chlorophyll A-B binding protein	112	30435	−0.03	−/3	11	53
N3 a	gi:115478691	LHCII type I	64	28053	0.101	−/3	7	56
N4	gi:115472785	LHCII type III	114	28817	0.025	−/2	12	62
N5	gi:115453971	LHCII type II	65	28534	−0.087	−/3	5	42
N6	gi:115458738	CP24	178	27043	0.062	−/2	12	50
O1	gi:149392661	PsbO	85	18990	−0.397	−/−	6	53
P1	gi:149392661	PsbO	81	18990	−0.397	−/−	5	53

a represent the spots which were identified with the cut-off of 200 ppm in spot No. column.

b represent the spots, which were identified by the MALDI-TOF/TOF, detail identification information of these spots was in the supplemental materials (Table S1).

c Qb (Segment) represents the plant species *Bruguiera gymnorhiza*.

d represent the number of identified peptides by PMF.

e represent the percentage of identified peptide coverage in total sequence of protein.

On the basis of protein identifications, identities of membrane complexes were speculated and displayed in Figure 4 and table 2. For example, the PSI complex exists in two forms: the PSI holo-complex and the PSIcore complex. The former contains the PSI core complex and LHCI complex. The heterodimer PsaA/B (A1, A2, E1 and E2, Figure 4) and the PsaE, PsaD proteins (A5, A6, E3 and E4, Figure 4) were identified in the form of the PSI holo-complex and the PSI core complex. The PsaE (A5, E3) and PsaD (A6, E4) proteins belong to the stromal ridge proteins of PSI. They are directly involved in the anchoring of flavodoxin and ferredoxin[1], acting important role in the photosynthesis efficiency. The recognition mechanism of the stromal ridge proteins was discussed below. The LHCI type III (A3, Figure 4) and small subunit PsaL (E5, Figure 4) were also identified through MS (Table 2). Some PSII supercomplexes were observed in front of the PSI complex in tobacco[36], as report these PSII supercomplexes consist of the PSII and LHCII, they were not observed in our study of rice. Similarly, the PSII complexes also exist in two forms: one is the PSII core complex, which was in adjacent with the cytochrome b6f complex at the MW of 309 kDa. We designated band E as PSIIcore/Cytb6f (Figure 1C and Figure 4). The other form lacks the CP43 subunit, and it was designated as CP43 Less PSII core (Figure 1 and Figure 4). We have identified the PSII reaction centre subunits D1(G3, H5, Figure 4) and D2 (G6, Figure 4) from the 2D gel. Furthermore, the core light-harvesting subunits CP43 (G1, Figure 4) and CP47 (H1, H2, H3 and H4, Figure 4) of PSII could also be observed. Some constituent subunits of Cytb6f complex, such as apocytochrome f (G2, Figure 4), cytochrome b6 (G4, Figure 4), cytochrome b6-f iron-sulfur (G5, Figure 4), and cytochrome b559 (G7 and H6, Figure 4) were also identified on 2D SDS-gels.

PSII complexes exist in two forms were also reported in other species[16], [37]. In this study, the spot M1(Figure 4) located on the right side of the gel was identified as CP43, which was also observed in barely[16]. It was supposed that the formation of the CP43 Less PSII core complex may result from this CP43 subunit detaching from the PSII core complex. During the photosynthesis, the subunits D1 and D2 of PSII could be subjected to the photodamage. The CP43-less PSII complex in stroma thylakoid regions was speculated to participate in the repair of the damaged PSII complex[38]. Therefore, our result showed the rice may also adopt the similar mechanism to repair the PSII system. In the CP43-less PSII complex, we could observe a serial, high abundant CP47 spots, which did not be displayed in the barely[16]. We identified all these spots as the CP47 with different MW. The different location of the CP47 in vertical row was supposed to be associated with its post-translational modification.

The ATPase complexes migrated as the forms of F_0_F_1_-ATPase and F_1_-ATPase complex (Figure 4), the ATPase α (B1, F1and F2, Figure 4), β (B2 and F3, Figure 4), and γ (F4, Figure 4) subunit were all identified from the gel.

The location of the PSI core complex was in front of the F1-ATPase complex with a MW of 379 kDa. The neighbouring distance of the PSI core complex and F1-ATPase complex in rice was very similar with previous report in the monocot barley[16]. The state of PSI core complex was speculated to be the transition or disassembly state[16].

The spots J1∼J4, L1∼L5 and N1∼N6 in Figure 4 were all identified as chlorophyll a/b-binding protein, which belong to the constituent parts of LHCII complexes. According to the masses of LHCII complexes, we may infer the identities of each protein complex at 108, 134 and 165 kDa in 1D BN-PAGE as monomeric, dimeric and trimeric forms of LHCII complexes. These chlorophyll a/b-binding subunits distributed as cluster range from 20 kDa to 35 kDa, which migrated in adjacent as previous report[21]. Spots L1, L2 and N1 were all identified as CP29 (Figure 4 and Table 2), suggesting post-translational modifications, such as phosphorylation, may exist in protein CP29. Because photosynthesis needs the collaboration of the PSI and PSII to ensure high efficiency, chlorophyll a/b-binding proteins would transform in the light-harvesting antenna and balance in two states[39], and it was reported that CP29 phosphorylation may be involved in the process[40]. Protein phosphorylations of other thylakoid membrane proteins were also reported in rice[41]. Several CP29 phosphorylation sites (Thr6, Thr16 and Thr32, and Ser102) have been identified by MS in the *Chlamydomonas reinhardtii* [40]. In the higher plant Arabidopsis, the Ser-Thr kinase STN7 involves in the phosphorylation of light harvesting protein CP29[42], while the STN8 involved in phosphorylation of the D1, D2, CP43 and PsbH proteins of PSII[43].

### The recognition mechanism of stromal ridge complexes in *Oryza sativa*

**Figure 5:**
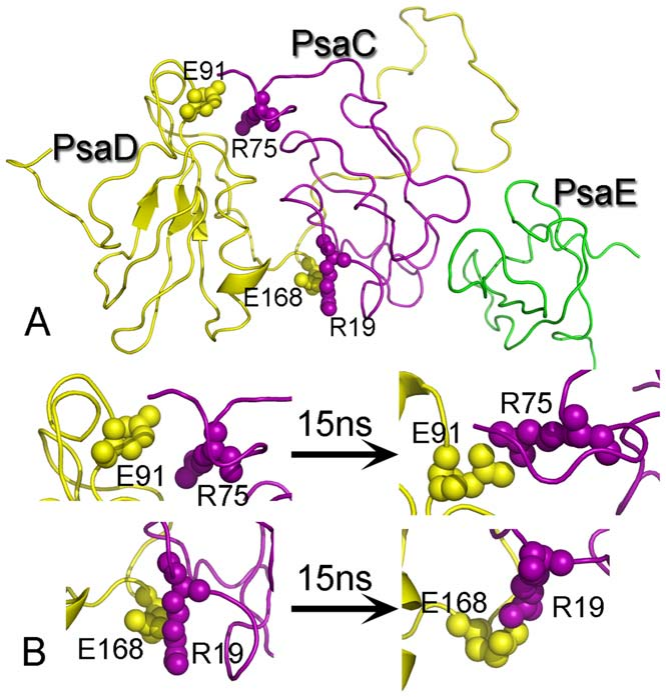
The modelled structure of the PsaC, PsaD and PsaE from rice (*Oryza sativa* L.). **A**: Two important salt bridge interactions were labelled on the model. B: The conformation change of the salt bridge during the 15 ns simulation.

**Figure 6:**
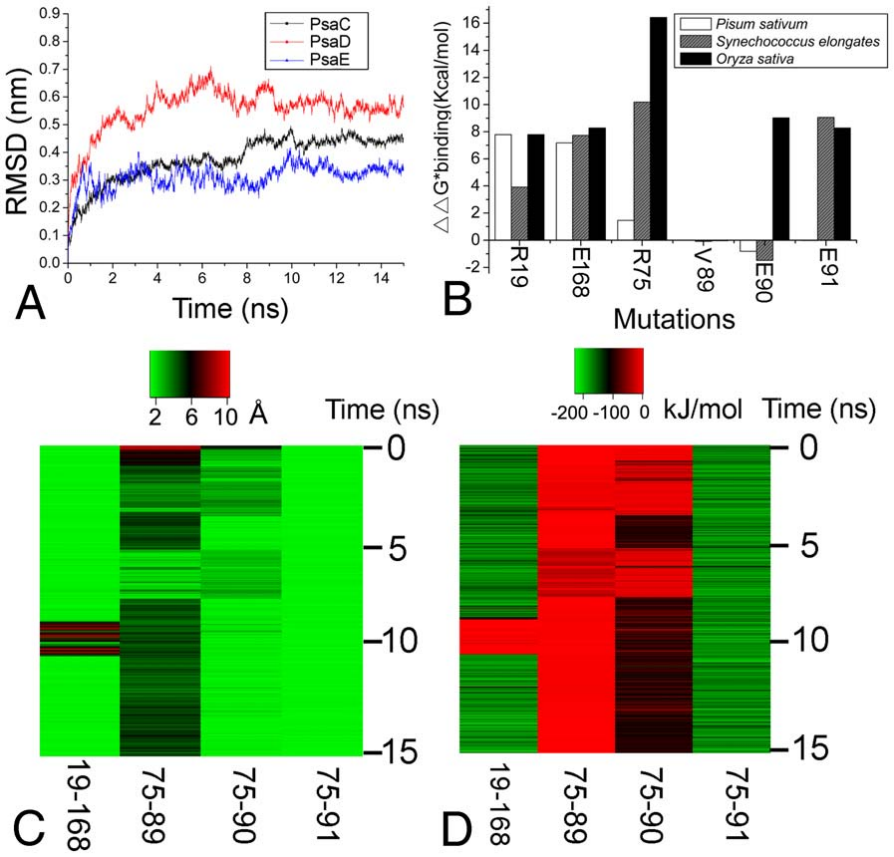
Characterization of stromal ridge complex in explicit solvent MD. A: The RMSD of the PsaC, PsaD and PsaE for the 15 ns MD run. B: Computational alanine scanning mutagenesis results for six single mutations from *Oryza sativa*, *Synechococcus elongates* and *Pisum sativum.* C: The closest atom distance in the residue pairs R19-E168, R75-V89, R75-E90, R75-E91. D: Interaction energy in the residue pairs R19-E168, R75-V89, R75-E90, R75-E91.

In PSI, the peripheral subunits PsaC, PsaD and PsaE contact closely, and function as anchoring of flavodoxin and ferredoxin[1]. These three subunits were called stromal ridge proteins. In our experiment, the stromal ridge proteins PsaD (A6 and E4 in Figure 4), PsaE (A5 and E3 in Figure 4) both in the PSI holo- and core- complex have been identified from rice. In previous studies, the spatial conformation of the stromal ridge complex have been solved from *Synechococcus elongates* (1JB0) [44] and *Pisum sativum* (2WSC) [45]. However, the stromal ridge conformation from *Oryza sativa* has not been reported. Thus, we modelled the stromal ridge complex in *Oryza sativa* based on previous structures [44], [45] (Figure 5A), and further refined the structure with a 15 ns explicit solvent MD simulation to study the recognition mechanism between these subunits. To assess the stability of the complex during the MD simulation, we calculated the structural drift relative to the structure just prior to the production of MD simulations. The root-mean square deviation (RMSD) of the equivalent Cα atoms relative to the reference structure was monitored along the trajectory and is shown Figure. 6A. The RMSD for PsaC, PsaD and PsaE reached a stable plateau after 9 ns simulations. A slightly higher RMSD value of about 5.2 Å was observed for PsaD during the simulation, as the loop of PsaD exhibiting great flexibility during the whole MD simulations. Furthermore, the RMSD for PsaE showed a quite lower value, about 3.0 Å, during the simulation.

Two important salt bridge interactions were observed both in the initial structure and the final refined structure (Figure 5B) in the simulation. In the first stage, we evaluated the contribution of the residues to the binding free energy using computational alanine scanning technique. A good agreement was found between the computational data of our modelled structure and the crystal structure directly derived from *Synechococcus elongatus* (1JBO) and *Pisum sativum* (2WSC). The high 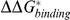 values were 7.79 and 16.42 kcal/mol for the polar residues R19 and R75 of PsaC when they were mutated to the alanine respectively, which suggested that these residues contribute greatly to the electrostatics interaction in the recognition process (Figure 6B). Besides, we also performed alanine scanning on the residues of E168, V89, E90, and E91 of PsaD (Figure 6B), which were all in a contact distance of 5 Å with R19 and R75 (Figure 6C). Results showed that E168 and E91 of PsaD mutation lead to a 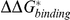 value rise of 8.27 and 8.27 kcal/mol respectively, indicating the residue pairs R19-E168 and R75-E91 might form two salt bridges in the stromal ridge of *Oryza sativa.* To the contrary, the mutation data on the residues V89 and E90 showed a lower change in 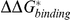 from *Synechococcus elongates* and *Pisum sativum*, suggested that these two residues might not be essential for the salt bridge formation.

In the second stage, we analyzed the closest interatomic distances between key polar residues obtained from the computational alanine scanning results. The distance of residues R19-E168 and residues R75-E91(Figure 5A and 5B) were found within a contact distance of 4.0 Å during the whole simulation time, which could explain the salt bridge interaction was stable during the MD simulation (Figure 6C). The result also shows the distance between the residues R75 and E90 was around 4 Å in the last half of the MD, while the R75 residue moved beyond a contact distance of 4 Å relative to the V89 during the whole MD simulation. This indicates the stability of the interaction of residue pairs R75-E90 and R75-V89 were not maintained during the whole MD simulation. To further elucidate the recognition mechanism of the stromal ridge from *Oryza sativa*, the short range (SR) coulombic interaction energy was calculated and displayed in Figure 6D. The SR coulombic interaction energy between residues R19 and E168 was constant with a value of ∼150 kJ/mol during the whole simulation time, excepting that there was a little fluctuation around 9 ns simulation time. These indicate the importance of the electrostatics energy in the residue pairs R19-E168. We then observed the SR coulombic interaction energy of the residue pairs R75-V89, R75-E90, and R75-E91. The energy value between residues R75 and E91 was constant with an average of ∼173 kJ/mol. These suggested the strong electrostatic interaction was mainly in the salt bridge of the residues R75 and E91. All the results showed these two salt bridges were essential to maintain the stability of stromal ridge complex in PSI from *Oryza sativa* L.

In this study, a new method designated “Double-Strip BN/SDS-PAGE” was introduced to separate thylakoid membrane protein complexes in the rice. Firstly, the effects of different detergents (NP-40, TritonX-100, and DDM) were evaluated for their abilities to dissolve membrane complexes from rice. Experimental data showed that the DDM-protein with a ratio of 4/1(g/g) could obtain the best result in dispersing membrane proteins in the form of “protein-detergent” micelles (Figure 1). Nine protein complexes with different MW in the range from 108 to 568 kDa could be observed on 1D native gels if stained by Coomassie blue R-250 (Figure 1C, Table 1). The identities of the membrane complexes could be characterized after 2D SDS-PAGE and MS identification, which included the holo- and core- PSI, the PSIIcore, Cytochrome b6/f, CP43-less of PSII core, the F_0_-F_1_ and F_1_ ATP synthase, the monomeric, dimeric, and trimeric forms of light harvest complex II (LHCII). Secondly, the subunit constitution of each native complex was separated by “Double-Strips BN/SDS-PAGE”. This new method clearly increased abundance of protein spots in 2D SDS-PAGE, and it enable protein spots to be excised more convenient as well as the protein subunit to be identified more ease by MS when compared with the traditional Single-strip BN/SDS-PAGE. The experiments in different condition also show the new method has some advantages over “Single-strip BN/SDS-PAGE” with high protein abundance, reproducibility and high resolution for separating thylakoid membranes not only in monocot but also in dicotyledon, such as *Oryza sativa*, *Spinacia oleracea* and *Cucurbita spp.* Finally, the spots excised from the 2D gel were identified by MALDI-TOF or MALDI-TOF/TOF, and the constituent subunits of each membrane complex were showed in a particular pattern in the 2D gel and these subunits in every membrane complex was exhibited natively in a vertical row on 2D SDS-PAGE. Interestingly, the stromal ridge subunits PsaD and PsaE were identified not only in the PSI holo- but also in core-complex. Up to now, the subunits interaction and conformation of the stromal ridge in rice has not been reported, we then used the molecular modelling method to construct the stromal ridge structure in rice. Some important interaction features between PsaC and PsaD has been predicated by bioinformatics, for instance, the basic residues R19 and R75 of PsaC could form salt bridge with the acidic reisidues E168 and E91 of PsaD. Furthermore, this interaction was further elucidated via the inter-molecular distance and interaction energy analysis. All together, our new approach developed for the resolution of thylakoid membrane complexes and the bioinformatics predications of subunit interactions in stromal ridge subcomplex would provide new insights into thylakoid membrane of rice.

## Methods

### Isolation of Chloroplasts

Chloroplasts were isolated from mature leaves of rice (*Oryza sativa* L.), spinach (*Spinacia oleracea*) and pumpkin (*Cucurita spp*) according to the method of Kugler[10] with the following modifications. Leaves (10 g) were powdered in liquid nitrogen and then homogenized in 100 ml ice-cold isolation buffer (330 mM sorbitol/50 mM HEPES/5 mM MgCl_2_/2 mM EDTA/2 mM NaF, pH 7.8). The homogenate was filtered through four layers of muslin. All subsequent steps were performed at 4°C. The filtrate was centrifuged at 200×g for 5 min to remove nuclei and cell debris. For sediment chloroplasts, the supernatant was centrifuged at 2,000×g for 10 min. The pellet containing crude chloroplasts was thoroughly suspended in 5 ml isolation buffer. To this end, crude chloroplast suspension was layered on top of a discontinuous sucrose gradient which consisted of 5 ml 60% (w/v) sucrose in the bottom, 8 ml 40% sucrose in the middle, and 5 ml 20% sucrose (all dissolved in the isolation buffer) on the top. After centrifugation at 2,500×g for 30 min, the dark green band at the interface of 40% to 60% sucrose which represents the intact chloroplasts was harvested and washed twice in isolation buffer by centrifugation at 3,000×g for 10 min. Finally the chloroplasts pellet was resuspended in isolation buffer supplemented with 20% glycerol (w/v) to a chlorophyll concentration of 1 mg/ml, which corresponded to a protein concentration of ∼10 mg/ml. The concentration of chlorophyll was determined using the Porra method[19] and the protein concentration was determined according to Bradford's method[20]. Aliquots of chloroplasts were frozen in liquid nitrogen and stored at −80°C until use.

### Solubilization of thylakoid membrane complexes

50 µl-aliquots of chloroplast suspension (50 µg chlorophyll, ∼500 µg protein) were used as starting material per slot of the blue-native gels. The chloroplasts were lysed in hypoosmotic buffer (50 mM HEPES, 2 mM MgCl_2_,1 mM EDTA, 1 mM NaF, 1 mM PMSF, pH 7.5) for 30 min at 4°C, and then centrifuged for 15 min at 15,000×g/4°C for collection of thylakoid membrane. In searching of the optimized condition for solubilization of membrane protein complexes, 50 µl of dodecylmaltoside solution made in ACA buffer (750 mM aminocaproic acid/50 mM Bis-Tris, pH 7.0/0.5 mM EDTA) at different concentrations (0.5, 1.0, 2.0, 3.0 and 4.0% [w/v]) was added to each sample fraction, thus thylakoid membrane were solubilized at varying DDM(*n*-dodecyl-β-D-maltoside)/protein ratio of 0.5/1, 1/1, 2/1, 3/1 and 4/1 (g/g). The sediment was pipetted until it became homogenate and incubated at 4°C for 20 min. After centrifugation at 15,000×g at 4°C for 30 min, the supernatant was supplemented with 10 µl of a Coomassie-blue solution (5% [w/v] Serva blue G in 750 mM aminocaproic acid) and loaded directly into one slot of the BN-gel. For detergent TritonX-100 and NP-40, we only selected the detergent/protein ratio of 4/1(g/g) in membrane dissolution.

### Blue native PAGE

Blue native PAGE was performed as described by Kugler[10]. Gels consisted of a separating (gradient of 5–12% acrylamide) and a stacking gel (4% acrylamide). Each lane was loaded with equal volume of the supernatant (as indicated above). As high molecular mass markers for native PAGE, a mixture of lyophilized standard proteins consisting of thyroglobulin, ferritin, catalase, lactate dehydrogenase and BSA (669, 440, 323, 140, 67 kDa, GE Healthcare, Amersham-Pharmacia, HMW native protein marker kit, 17-0445-01,UK) was applied. The electrophoresis was carried out at 4°C. Protein complexes in the BN-gel can be visualized directly or stained by Coomassie Blue when the electrophoresis finished.

### Single- and double-strips SDS-PAGE

Each lane excised from 1D BN-gel, which was loaded with aliquots of chloroplast (50 µg chlorophyll), was incubated in 50 ml denaturing solution [4% SDS (w/v), 100 mM Tris, 5% β-mercaptoethanol (v/v), 10% glycerol (v/v); pH 6.8] for 30 min at 37°C and then rinsed in distilled water to remove excess β-mercaptoethanol, which is a strong inhibitor for the polymerization of acrylamide[21]. For 2D double-strips SDS-PAGE, two strips with 1 mm thickness were cut from two adjacent lanes, and then they were stacked on each other (50 µg +50 µg chlorophyll) and placed between two glass plates using 1.5 mm spacers (Figure 2). The SDS-gel consisted of a 12.5% uniform separating gel and a 4% stacking gel with the dimensions of 0.15×20×19 cm. A lower melting agarose solution [1% (w/v) agarose, 192 mM glycine, 0.1% SDS and 25 mM Tris pH 6.8] was used to fill in the space between the overlapped BN-gel strips and stacking gel. For 2D single-strip SDS-PAGE, same concentrations of acrylamide in separating and stacking gel were used. To compare with same proteins loading in Single- and double-strips SDS-PAGE, 100 µg chlorophyll was loaded into one slot in 1D BN-PAGE, and only one lane was used for Single-strip BN/SDS-PAGE. As for double-strips SDS-PAGE, the two lanes (each lane was loaded with 50 µg chlorophyll in 1D BN-PAGE) were overlapped in 2D SDS-PAGE. After finishing electrophoresis, gels were stained with 0.25% Coomassie blue R-250 in 40% (v/v) methanol and 10% (v/v) acetic acid, destained with 5% (v/v) methanol and 7% (v/v) acetic acid until the background was clear, then scanned.

### Identification of proteins by mass spectrometry

Protein spots were excised from gels and digested in-gel using trypsin (Promega, Madison, WI). Each dried peptide mixture was dissolved with a volume of 50% ACN/0.1% TFA according to its relative abundance in the gel. Millipore C18 ZipTips were used (Millipore, Bedford, MA, USA) to remove salts and detergents. Bound peptides were eluted from ZipTip with approximately 3 µl of 60% methanol/3% formic acid. 0.5 µl sample solution or calibration standard was then mixed with equal volume of CHCA (a-cyano-4-hydroxycinnamic acid) matrix (10 mg/ml CHCA in 50% ACN/0.1% TFA) and spotted onto a freshly cleaned target plate. After air drying, the crystallized spots were processed with MALDI-TOF mass spectrometer (Voyager-DE STR, Applied Biosystems) operating in positive reflection mode under the conditions of 20 kV accelerating voltage, 72% grid voltage, 300 ns extraction delay time, 1.12 mirror voltage ratio and 0.002% guide wire. Mass peaks were collected by 200 laser shots per spectrum with a mass range from 1000 to 5000 Da. The external calibration was performed before sample analysis, using a peptide standard kit (Applied Biosystems) in which included angiotensin I ([M+H]^+^ = 1296.6853), ACTH 1-17 ([M+H]^+^ = 2093.0867), ACTH 18-39 ([M+H]^+^ = 2465.1989) and ACTH 7-38 ([M+H]^+^ = 3657.9294). The calibration was processed by Data Explorer version 4.0. Data acquired by MALDI-TOF were used for protein identification by peptide mass fingerprinting (PMF). They were opened by Data Explorer and searched through Mascot Wizard (Matrix Science, v. 1.2.0.0) against the publicly available NCBInr protein database, taxonomy *Oryza sativa* (Date 2011-01-31, 134548 sequences). The searching parameters were set up as follows: 100 ppm of mass tolerance (only the spots A1, A4, B1, B2, G5 and N3 with 200 ppm in Table 2); allowing for up to one missed cleavage site; carbamidomethyl (C) as fix modification; oxidation (M) as variable modification; monoisotopic as mass values. Results with scores over the significant threshold (p<0.05) were considered credible.

As a few spots, such as A2, E1, E5, G6, I1, J2, K2, L1, L2 and N1 (Table 2), cannot be identified by PMF, thus they were further analyzed by MALDI-TOF/TOF (4800 Plus Analyzer, Applied Biosystems). After calibration, parent mass peaks were scanned in 1000 laser shots with a mass range of 800∼4000 Da. The minimum signal to noise ratio was 10. Five parent mass peaks with most intensity were picked out for tandem TOF/TOF analysis, each with 1500 laser shots. Spectra combined mass and mass/mass were searched against an NCBInr protein database, taxonomy *Oryza sativa* (date 2008-06-03) by GPS Explorer™ Workstation (Applied Biosystems). The searching parameters were set as follows: carbamidomethylation(C) and oxidation (M) as variable modifications, up to one missed cleavage, precursor ion tolerance at 200 ppm, and fragment ion tolerance at 0.3 Da and peptide charge of 1+. Protein hits with protein score C. I.% (confident identification percentage, based on combined mass and mass/mass spectra) over 95 were reserved. Most identified proteins also have total ion score C. I.% (based on mass/mass spectra) over 95.

### The atomic coordinates of the complex PsaC, PsaD and PsaE from *Oryza sativa*

The amino acid sequence of the PsaC, PsaD and PsaE from rice was obtained from the NCBI (ID: gi|11466848, gi|115477831 and gi|34394725). For generating atomic coordinates of the stromal ridge complex, in the first homology modelling step, template structures related to the PsaC, PsaD, PsaE of rice were searched against the whole Protein Data Bank[22] using the Blast algorithm. And then, we modelled the complex of PsaC, PsaD and PsaE from rice using the photysystem I crystal structure from *Pisum sativum* (PDB id: 2WSC, sequence identity 89.08%) as a template through the SWISS-MODEL server[23].

To introduce the solvation effects that may affect the interaction between the PsaC, PsaD and PsaE of rice, we run 15 ns MD simulation to further refine and validate the model. The homology modelling combined with the MD simulation are useful to assess the structure quality[24]. These MD simulations were performed with the GROMACS 4.5 software package[25] using the OPLS force field[26] and the SPC216 water model. The protonation state of ionizable groups was chosen to correspond to pH 7.0. Counterions were added to compensate the net charge of the system. The initial structure of the complex was immersed in a periodic water box. The electrostatic interactions were calculated using the Particle-mesh Ewald (PME) algorithm[27], and the van der Waals forces were treated with a cutoff distance of 10 Å. After 3000 steps of energy minimization using a steepest descent method, the system was subject to 100 ps of equilibration at 300 K and normal pressure, using harmonic position restraints with a force constant of 1000 kJ mol^-1^ nm^−2^. The system was coupled to an external bath by the Berendsen pressure and temperature coupling method[28]. The 15 ns production run was performed under the same conditions except that all position restraints were removed. The results were analyzed using the standard software tools provided by the GROMACS 4.5 package[25]. Visualization and manipulation of the conformations was performed using the programs PyMOL 0.99 (http://www.pymol.org/). The distance and energy analysis of the nonpolar residue-pairs was completed using the R statistical software package[29].

### Calculation of Binding Free Energies by MM-GBSA

To further evaluate the binding ability of the three stromal ridge subunits (PsaC, PsaD, and PsaE), we use the Amber11 program for binding free energy calculation. The MM-GBSA approach show the advantage to estimate the binding free energy of the complex[30], [31]. In this study, the implicit generalized Born solvation model was used (igb = 2). The temperature was set to 300 K. Non-bonded interactions were cut off at a distance of 12 Å. The ff99 force field (Parm99)[32] was applied throughout the energy minimization and MD simulations.

In the MM-GBSA implementation of Amber 11, the binding free energy of A+B→AB is calculated using the following thermodynamic cycle:
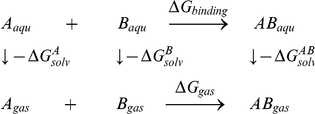


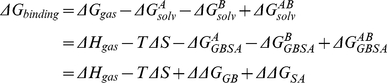


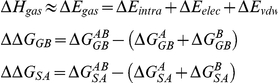



Where T is the temperature, S is the solute entropy, 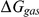 is the interaction energy between A and B in the gas phase, and 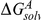, 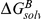, and 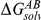 are the solvation free energies of A, B, and AB, which are estimated using a GB surface area (GBSA) method [31], [33]. That is, 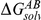  = 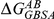 + 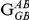 + 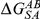, and so forth. 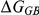 and 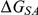 are the electrostatic and nonpolar terms, respectively. The bond, angle, and torsion energies constitute the intramolecular energy 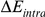 of the complex, while 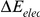 and 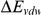 represent the receptor-ligand electrostatic and van der Waals interactions, respectively. We refer to 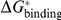 for 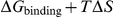 in the discussion. To verify the quality and validity of the complexes, the relative binding free energy 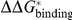 was calculated using MM-GBSA for post processing snapshots from the MD trajectories. The computational alanine scanning method in MM-GBSA was used to evaluate the important of the residues contributing to the binding ability. The key residues were mutated to alanine and subsequently the difference in the binding free energies between mutated and wild-type complexes was calculated based on the MM-GBSA approach. The calculated results were compared with the experimental data.

## Supporting Information

Figure S1 Comparison of patterns derived from different protein loading between the Double-Strips and Single-Strip BN/SDS PAGE. D1-1∼D1-4: The pattern of Double-Strips BN/SDS-PAGE with two 1D BN-Gel strips. S2-1∼ S2-4: The pattern of Single-Strip BN/SDS-PAGE with one 1D BN-Gel strip. Each strip excised from 1D BN-Gel lane loaded with 50 µg chlorophyll and the membrane dissolved at DDM/protein ratio of 4/1. Each set of experiment was repeated four times.
(TIF)

Figure S2 Comparison of patterns derived from equal protein loading between the Double-Strips and Single-Strip BN/SDS PAGE. D1-1∼D1-4: The patterns of Double-Strips BN/SDS-PAGE with two 1D BN-Gel strips each loaded with 50 µg chlorophyll dissolved at DDM/protein ratio of 4/1. S1-1∼ S1- 4: The patterns of Single-Strip BN/SDS-PAGE with one 1D BN-Gel strip loaded with 100 µg chlorophyll dissolved at DDM/protein ratio of 4/1. S3-1∼ S3-4: The patterns of Single-Strip BN/SDS-PAGE with one 1D BN-Gel strip loaded with 100 µg chlorophyll dissolved at DDM/protein ratio of 2/1. Each set of experiment was repeated four times.
(TIF)

Figure S3 The mass spectra for the proteins identified with the mass cut off by100 ppm listed in the Table 2 of the main text.
(TIF)

Table S1 Thylakoid membrane proteins identified from rice (*Oryza sativa* L.) by MALDI-TOF/TOF.
(DOC)
