# Supplementary figures and images for: Isolation of Thylakoid Membrane Complexes from Rice by a New Double-Strips BN/SDS-PAGE and Bioinformatics Prediction of Stromal Ridge Subunits Interaction
Source: PLoS One. 2011 May 26;6(5):e20342. doi: 10.1371/journal.pone.0020342 (PMC3102703; doi:10.1371/journal.pone.0020342)

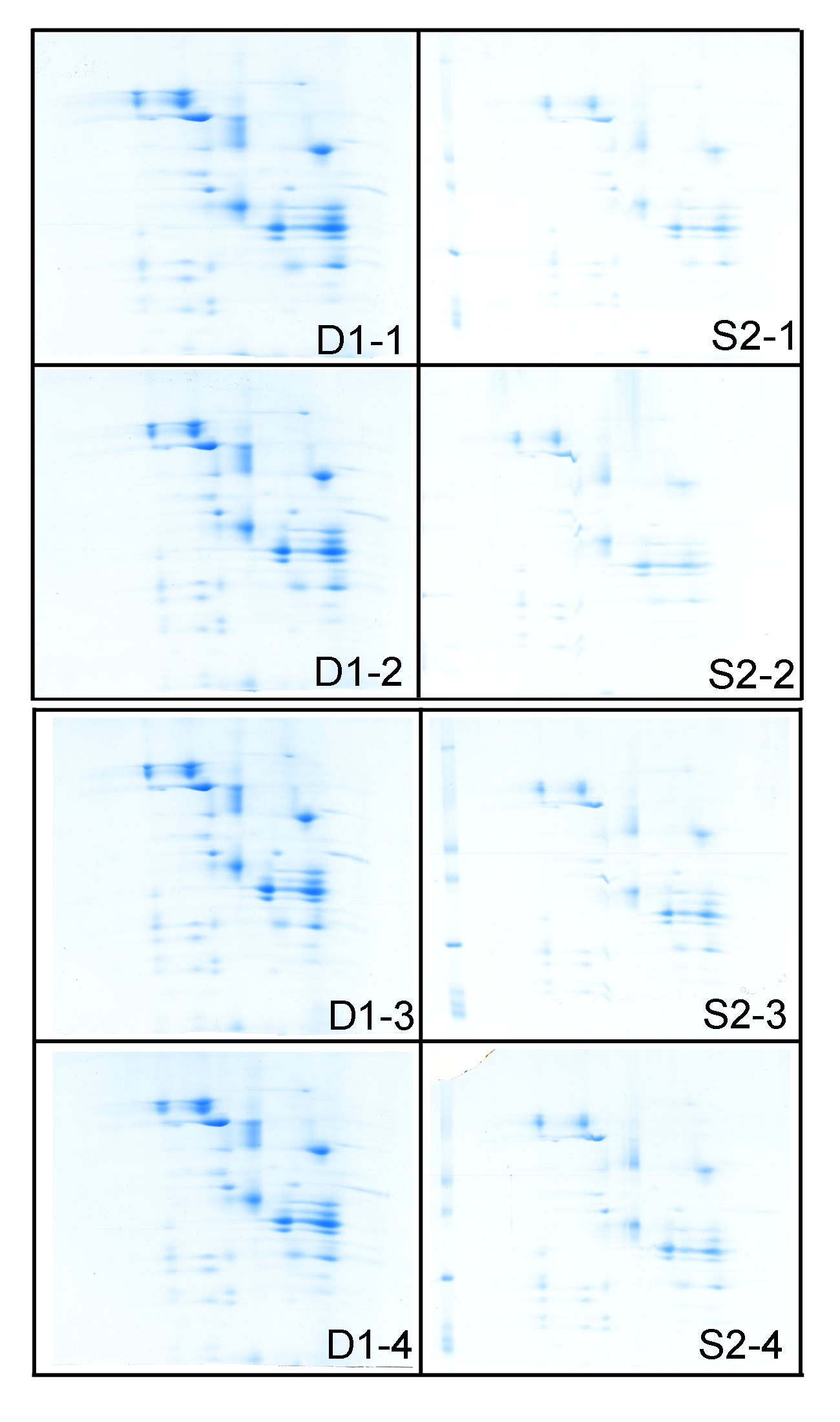

Supplement: Figure S1 — Comparison of patterns derived from different protein loading between the Double-Strips and Single-Strip BN/SDS PAGE. D1-1∼D1-4: The pattern of Double-Strips BN/SDS-PAGE with two 1D BN-Gel strips. S2-1∼ S2-4: The pattern of Single-Strip BN/SDS-PAGE with one 1D BN-Gel strip. Each strip excised from 1D BN-Gel lane loaded with 50 µg chlorophyll and the membrane dissolved at DDM/protein ratio of 4/1. Each set of experiment was repeated four times. (TIF) [file pone.0020342.s001.tif]

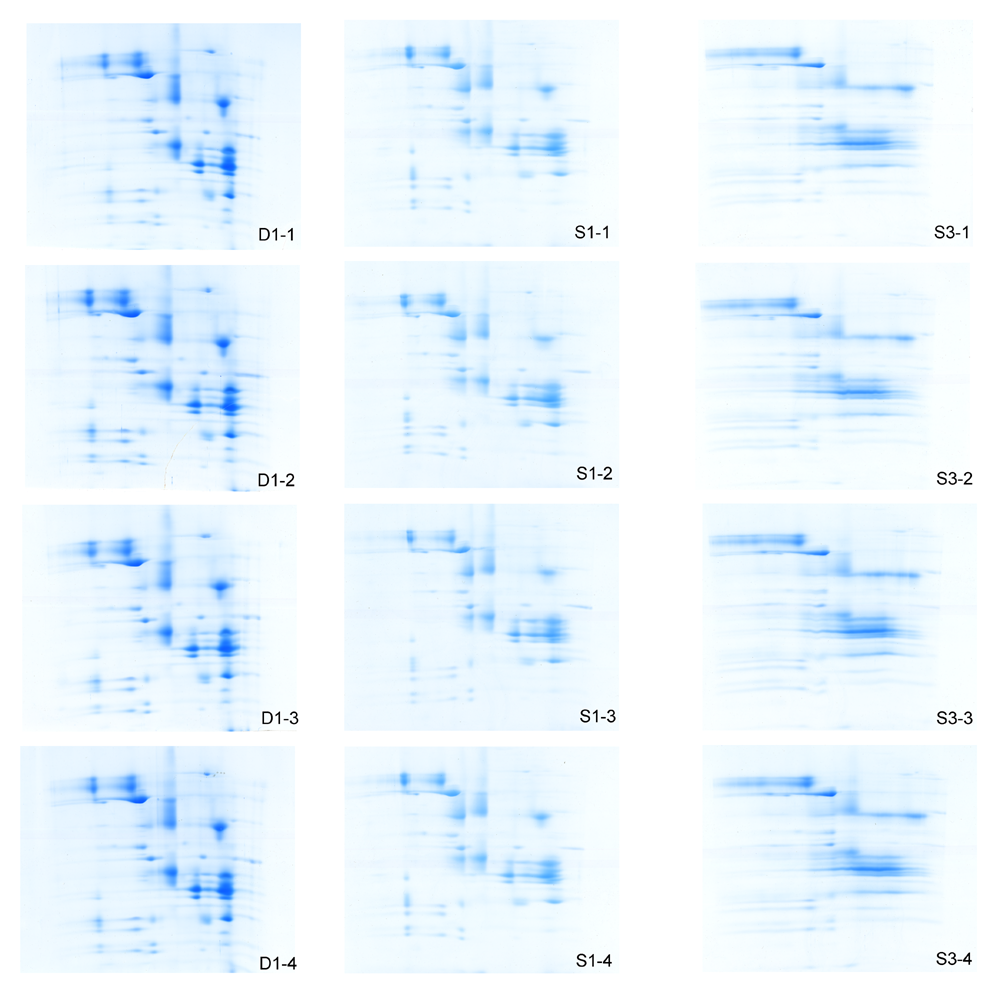

Supplement: Figure S2 — Comparison of patterns derived from equal protein loading between the Double-Strips and Single-Strip BN/SDS PAGE. D1-1∼D1-4: The patterns of Double-Strips BN/SDS-PAGE with two 1D BN-Gel strips each loaded with 50 µg chlorophyll dissolved at DDM/protein ratio of 4/1. S1-1∼ S1- 4: The patterns of Single-Strip BN/SDS-PAGE with one 1D BN-Gel strip loaded with 100 µg chlorophyll dissolved at DDM/protein ratio of 4/1. S3-1∼ S3-4: The patterns of Single-Strip BN/SDS-PAGE with one 1D BN-Gel strip loaded with 100 µg chlorophyll dissolved at DDM/protein ratio of 2/1. Each set of experiment was repeated four times. (TIF) [file pone.0020342.s002.tif]

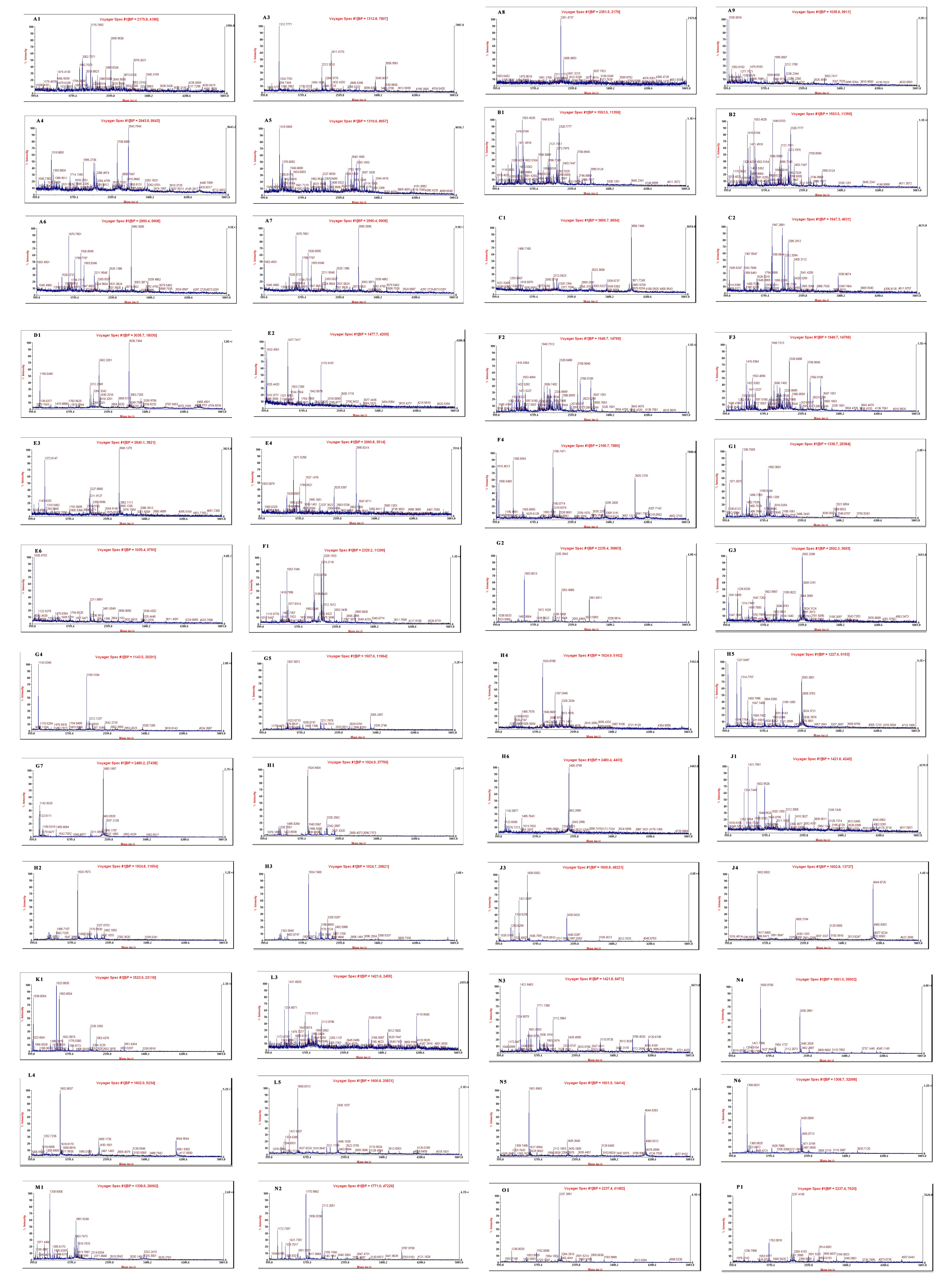

Supplement: Figure S3 — The mass spectra for the proteins identified with the mass cut off by100 ppm listed in the Table 2 of the main text. (TIF) [file pone.0020342.s003.tif]
