# Supplementary material for: Isolation of Thylakoid Membrane Complexes from Rice by a New Double-Strips BN/SDS-PAGE and Bioinformatics Prediction of Stromal Ridge Subunits Interaction
Source: PLoS One. 2011 May 26;6(5):e20342. doi: 10.1371/journal.pone.0020342 (PMC3102703; doi:10.1371/journal.pone.0020342)
Supplement: Table S1 — Thylakoid membrane proteins identified from rice (Oryza sativa L.) by MALDI-TOF/TOF. (DOC) [file pone.0020342.s004.doc]

Table S1. Thylakoid membrane proteins identified from rice (*Oryza sativa* L.) by MALDI-TOF/TOF

| Spot No. | Accession number | Protein description | PI | Protein Score | Protein Score C. I. % | Total Ion Score | Total Ion  C. I. % |
| --- | --- | --- | --- | --- | --- | --- | --- |
| A2 | gi:20146741 | PsaA | 6.6 | 135 | 100 | 131 | 100 |
| E1 | gi:20146741 | PsaA | 6.6 | 135 | 100 | 131 | 100 |
| E5 | gi:131225 | PSI-L | 9.52 | 108 | 100 | 100 | 100 |
| G6 | gi:8131597 | Qb(Segment)*^a^* | 4.71 | 256 | 100 | 245 | 100 |
| I1 | gi:218155 | chloroplastic aldolase | 7.6 | 69 | 99.336 | 60 | 99.964 |
| J2 | gi:115438250 | LHCII type I | 5.29 | 85 | 99.983 | 74 | 99.998 |
| K2 | gi:115458738 | CP24 | 6.75 | 65 | 98.333 | 57 | 99.868 |
| L1 | gi:115472753 | CP29 | 5.33 | 80 | 99.948 | 72 | 99.996 |
| L2 | gi:115472753 | CP29 | 5.33 | 79 | 99.939 | 70 | 99.995 |
| N1 | gi:115472753 | CP29 | 5.33 | 182 | 100 | 170 | 100 |

*^a^* represent the species *Bruguiera gymnorhiza*.
